# A Large Scale Molecular Hessian Database for Optimizing Reactive Machine Learning Interatomic Potentials

**DOI:** 10.1038/s41597-025-06350-5

**Published:** 2025-12-04

**Authors:** Taoyong Cui, Yunhong Han, Haojun Jia, Chenru Duan, Qiyuan Zhao

**Affiliations:** Deep Principle Inc., Cambridge, MA 02139 USA

**Keywords:** Computational chemistry, Chemical physics

## Abstract

Transition-state (TS) characterization underpins reaction modeling but conventional DFT is costly. Machine-learning interatomic potentials (MLIPs) promise quantum-level accuracy at lower cost, yet, lacking large-scale Hessian data, most are pretrained only on energies and forces, limiting TS optimization. We present HORM, the largest quantum-chemistry Hessian dataset for reactive systems: 1.84 million matrices at the *ω*B97x/6-31G(d) level. To exploit second-order information efficiently, we propose Hessian-informed training with stochastic row sampling, which controls the computational overhead of incorporating Hessians. Across diverse MLIP architectures and force-learning schemes, HORM yields up to 63% lower Hessian mean absolute error and up to 200× improvement in TS-search efficiency versus counterparts trained without Hessians. HORM thus fills critical data and methodological gaps, enabling more accurate, robust reactive MLIPs and scalable exploration of reaction networks.

## Background & Summary

Computational transition state (TS) characterization is essential for elucidating reaction mechanisms, differentiating between competing reaction pathways, and predicting reaction kinetics and thermodynamics, making it an indispensable tool in computational chemistry for a wide range of applications^1–6^. Traditional computational TS search relies on costly density functional theory (DFT) calculations to evaluate energy and forces across large reactive spaces, along with Hessian calculations for saddle point optimization^7–9^. However, the exponential growth of chemical space in modern drug discovery and materials science makes such costly calculations increasingly inadequate for meeting the demands of large-scale reaction predictions^10^. To address the high computational demands, machine learning (ML) approaches have emerged as powerful accelerators by significantly reducing dependence on DFT calculations^5,10–13^. One of the most promising tools is machine learning interatomic potentials (MLIPs), which accurately characterize potential energy surfaces (PES) at low computational cost^14,15^. By enabling efficient evaluation of energy landscapes, interatomic forces, and Hessian matrices, MLIPs naturally integrate with physics-based transition state search algorithms.

MLIPs have emerged as powerful tools for predicting energies and forces with quantum-mechanical accuracy, providing efficient approximations of potential energy surfaces from atomic configurations defined by element types and atomic positions^15–21^. These models typically follow one of two strategies. Autograd-based models predict total energies and compute forces as analytic gradients, thereby guaranteeing energy conservation and physically consistent force symmetries, though at the cost of increased training and inference time. Direct-force models, by contrast, predict energies and forces separately, offering improved efficiency but often producing non-conservative and asymmetric forces that can compromise accuracy in downstream applications such as molecular dynamics or transition state (TS) searches^22^. Recent state-of-the-art architectures illustrate both paradigms: EquiformerV2^23^ and Orb-V2^24^ exemplify direct-force models, while MACE^25,26^, Orb-V3^27^, and MatterSim^28^ represent autograd-based approaches. All of these models achieve remarkable accuracy on equilibrium systems; however, extending such success to reactive systems involving bond-breaking and bond-forming remains a significant challenge due to the broader chemical diversity and more complex regions of the potential energy surface. Schreiner *et al*. showed that PaiNN can identify transition states across diverse reactions when trained on the Transition1x dataset^29^. Yuan *et al*. further demonstrated that fine-tuning an equivariant message-passing neural network on Transition1x can enhance TS optimization, even without access to second-order (Hessian) information^30^. More recently, Anstine *et al*. introduced AIMNet2 series models, expanding the chemical domain of reactive MLIPs from main-group elements to transition metal catalysis by training on diverse datasets^31–33^. However, a recent benchmark study evaluating universal MLIPs in an end-to-end TS search workflow reveals that many models—especially models that directly predict forces—struggle with transition state optimization due to inaccurate and even asymmetric Hessian predictions, a limitation rooted in training solely on energies and forces without access to second-order information^34^.

The exclusion of Hessian matrices in most MLIP development arises from two central challenges: the lack of available datasets and the high cost of incorporating second-order information during training. On the data side, nearly all widely used MLIP datasets focus exclusively on energies and forces, with Hessian information extremely rare. For example, Transition-1x^35^, a benchmark dataset for reactive MLIP training, and the recently released large-scale OMol25^36^, containing 10M and 100M geometries respectively, do not include Hessians. One of the few exceptions is Hessian-QM9, which provides DFT-computed Hessians for 41,645 equilibrium molecules at the *ω*B97x/6-31G(d) level^37^. While valuable for vibrational property prediction, its restriction to equilibrium structures and limited chemical diversity reduces its relevance for reactive systems, highlighting the need for broader datasets that capture non-equilibrium geometries critical for reaction mechanisms and transition state optimization. Alongside these data limitations, methodological hurdles further complicate the use of Hessians in MLIP training. For equilibrium structures, several approaches have emerged: Hessian-QM9 has been used to train E(3)-equivariant message-passing models to improve vibrational property prediction^38^, while DetaNet introduced a partitioned tensor framework decomposing Hessians into atomic and interatomic components for scalable learning^39^. Building on this, EnviroDetaNet incorporated environmental context, yielding a 42% accuracy improvement in Hessian prediction and enabling more precise spectral modeling^40^. For non-equilibrium structures, most methods infer Hessians indirectly from derivatives of force predictions. A recent advance by Amin *et al*.^41^ employed knowledge distillation, allowing a student MLIP to learn Hessian representations from foundation models such as MACE-OFF23^25^, thereby improving energy and force accuracy without requiring explicit DFT Hessians. Meanwhile, transition state optimization studies have underscored the importance of Hessians derived from MLIPs in achieving reliable saddle point convergence^30,34^. Although second-order information can be partially captured through force-based training, the absence of explicit Hessian supervision often leads to inaccurate and asymmetric predictions, limiting the robustness of current approaches.

In this work, we introduced HORM (Hessian dataset for Optimizing Reactive MLIP), the largest quantum chemistry database of reactive systems to date, comprising 1.84 million Hessians computed at the *ω*B97x/6-31G(d) level of theory. The geometries are sampled from two reaction databases, transition-1x and RGD1, covering C, H, O, and N containing molecules with up to 10 heavy atoms. By addressing a critical data gap, HORM enables the robust training of reactive machine learning interatomic potentials (MLIPs) with significantly improved Hessian quality—applicable to both direct-force and autograd-based architectures. Notably, when Hessian constraints are used to enforce force symmetry, a representative direct-force model, EquiformerV2, exhibits a 30 to 200 times improvement in Hessian accuracy and TS search performance, respectively. This finding highlights a promising direction to overcome key limitations of direct-force MLIPs.

## Methods

### Dataset Composition

The geometries in the HORM dataset are sampled from two reactive datasets, Transition1x^35^ and RGD1^42^. The reactions of the two datasets are generated by unbiased graph-based enumeration. In Transition1x, no more than six bond changes are allowed, while in RGD1, up to two bonds can break and two bonds can form (e.g., dehydration reaction, keto-enol tautomerization). Among the 10,073 reactions in Transition1x, we adopt a previously established data split based on reaction identities, assigning 9,000 reactions to the training set and the remaining 1,073 to the validation set^10^. From these, 1,725,362 geometries corresponding to the training reactions and 50,844 geometries from the validation reactions are included in the HORM dataset, representing 20% and 5% of the available geometries from each split, respectively. To sample non-equilibrium geometries from the RGD1 dataset, we leverage reaction pathways generated via IRC calculations performed at the GFN2-xTB level of theory. From approximately 950,000 available reactions, we randomly selected 80,000 and sampled up to 15 geometries per reaction along their IRC results. From this pool, 60,000 geometries were randomly chosen to constitute the final RGD1 subset. It is worth highlighting that the initial geometries in the two datasets are retained, coming from DFT-level (wb97x/6-31G(d)) NEB optimizations and GFN2-xTB level IRC calculations, respectively. It is worth noting that, because HORM is derived from the Transition1x and RGD1 datasets, it contains only C, H, O, and N species with up to ten heavy atoms, limiting its direct applicability to larger molecules or more elementally diverse chemical spaces.

### DFT calculations

All DFT calculations were carried out using the GPU4PYSCF v1.3.0^43^. The *ω*B97x functional and the 6-31G(d) basis set were employed to create data compatible with the original Transition1x dataset, commonly used ANI-1x and ANI-2x datasets, and the recently released Hessian-QM9 dataset. All molecules in the set are neutral with multiplicity equal to 1. The self-consistent field (SCF) cycle was deemed converged when the change in total energy was less than 1e-10 Hartree and the orbital-gradient norm was less than 1e-5, which are default settings of GPU4PYSCF v1.3.0. The hessian calculations are performed with the built-in “Hessian()” object in PYSCF.

### MLIP Training

An energy and force (E-F) loss function that combines errors in both energy and force predictions is commonly used for training MLIPs. The total loss function is defined as:

1 $${{\mathcal{L}}}_{{\rm{EF}}}=\alpha \parallel E-\widehat{E}\parallel +\frac{\beta }{N}\mathop{\sum }\limits_{i=0}^{N-1}\left\Vert {{\bf{F}}}_{i}-\left(-\frac{\partial \widehat{E}}{\partial {{\bf{r}}}_{i}}\right)\right\Vert ,$$

where N is the total number of atoms and *α* and *β* are the weights assigned to the energy and force losses, respectively.

Given a dataset ${\mathcal{D}}={\{({{\bf{z}}}_{i},{{\bf{r}}}_{i},{U}_{i},{{\bf{F}}}_{i},{{\bf{H}}}_{i})\}}_{i=1}^{N}$ consisting of *N* molecular structures with atomic numbers, positions, and DFT-calculated energy, force, and Hessian matrix, the MLIP (*ϕ*) is trained to simultaneously match all three DFT-computed quantities.

To mitigate the ${\mathcal{O}}({N}^{2})$ computational cost of full Hessian computation via automatic differentiation, we implement a stochastic row-sampling strategy. In each training epoch, *s* rows ${{\mathcal{J}}}_{i}=\{{j}_{1},\ldots ,{j}_{s}\}\subset \{1,\ldots ,3N\}$ are randomly selected for each molecular structure in each epoch, where each index corresponds to an atomic coordinate. This sampling reduces the Hessian computation complexity to ${\mathcal{O}}(s)$ while maintaining learning efficacy. The modified loss function becomes:

2 $${\mathcal{L}}(\phi )={{\mathcal{L}}}_{{\rm{EF}}}(\phi )+\gamma {{\mathbb{E}}}_{{{\mathcal{J}}}_{i} \sim {{\mathcal{U}}}_{s}}\left(\frac{1}{s}\sum _{j\in {{\mathcal{J}}}_{i}}{\left\Vert {{\bf{H}}}_{i}^{(j)}+\partial {F}_{\phi }^{(j)}({{\bf{z}}}_{i},{{\bf{r}}}_{i})/\partial {\bf{r}}\right\Vert }_{2}\right),$$

where ${{\mathcal{U}}}_{s}$ denotes a uniform distribution (1,3N) over subsets of *s* rows from the Hessian, *γ* is the weight of the Hessian loss, ${{\bf{H}}}_{i}^{(j)}$ is the *j*-th row of the reference Hessian of molecule *i*, and ${F}_{\phi }^{(j)}$ is the MLIP-predicted force component.

Row extraction is efficiently performed via vector-Jacobian products (VJPs)^41^: given a MLIP force ${\bf{F}}:{{\mathbb{R}}}^{3N}\to {{\mathbb{R}}}^{3N}$ with Jacobian **H**, selected rows ${{\bf{P}}}_{{\mathcal{J}}}{\bf{H}}\in {{\mathbb{R}}}^{s\times 3N}$ are computed using batched VJPs **v**^⊤^∂_**r**_**F**, where ${{\bf{P}}}_{{\mathcal{J}}}={[{{\bf{e}}}_{{j}_{1}},\ldots ,{{\bf{e}}}_{{j}_{s}}]}^{\top }$ contains one-hot vectors **v**_*k*_. A vmap-optimized implementation further accelerates this process by avoiding explicit construction of the full Hessian matrix.

Including Hessian data inevitably leads to an increase in training time, with the average epoch becoming approximately 76% longer due to the additional cost of second-order derivative supervision. Nevertheless, the inference efficiency remains unaffected, as the model architecture and evaluation procedure are identical regardless of whether Hessian information is included during training.

### Transition State Search and Verification

The TS search and validation workflow follows the MLIP-based protocol established in our previous work^34^ and proceeds in four steps. First, reactant and product geometries are optimized at the target MLIP level. Second, a growing-string method (GSM) calculation is initiated from these MLIP-optimized endpoints to construct a minimum-energy path (MEP), with energies and gradients evaluated by the same MLIP. Third, the highest-energy node along the MEP (the TS initial guess) is refined using a Hessian-informed restricted-step rational-function-optimization (RS-I-RFO) algorithm; the Hessian is assembled from derivatives of the MLIP-predicted forces and guides the TS refinement. Fourth, the refined saddle is checked by frequency analysis (single imaginary frequency) and verified by intrinsic reaction coordinate (IRC) integration in both directions; the forward and reverse IRC endpoints are compared to the input reactant and product based on the atom connectivity. A TS is labeled “intended” only if both IRC endpoints match the corresponding input endpoints; otherwise, it is labeled “unintended.”

We applied this workflow to the same 960 validation reactions used in the prior study to avoid data leakage^34^. Reported quantities include overall TS-finding success rate and intended-TS rate, absolute barrier error $\Delta {E}^{\ddagger }={E}_{{\rm{MLIP}}}^{\ddagger }-{E}_{{\rm{DFT}}}^{\ddagger }$, TS-geometry RMSD between MLIP- and DFT-optimized TSs. It is worth noting that the reactant and product geometries are directly inherited from the Transition1x dataset to ensure consistency with previous studies. A more comprehensive conformational sampling strategy could further improve the success rate and intended rate of TS searches, and we highlight this as a potential extension for future work.

## Data Records

All the data is available from Zenodo^44^. The HORM dataset is distributed in the form of three LMDB databases, each containing molecular geometries and associated quantum chemical properties computed at the *ω*B97X/6-31G(d) level of theory. The dataset is organized into one training set and two evaluation subsets as follows:

- ts1x_hess_train.lmdb - Training set that contains 1,725,362 geometries sampled from the Transition1x dataset.
- ts1x-val.lmdb - In-distribution validation set that contains 50,844 geometries sampled from Transition1x.
- RGD1.lmdb - Out-of-distribution test set that contains 60,000 geometries sampled from the RGD1 dataset.

Each LMDB entry corresponds to a single molecular geometry and contains information of its chemical composition, coordinates of atoms, computed energy, forces, and Hessian (Table 1). The full dataset comprises over 1.8 million off-equilibrium molecular structures sampled along reaction pathways. Structures from the Transition1x-derived subsets (ts1x_hess_train.lmdb and ts1x-val.lmdb) include molecules with up to seven heavy atoms and represent geometries sampled during DFT-level nudged elastic band (NEB) calculations. The RGD1.lmdb subset features geometries sampled from IRC pathways of the RGD1 dataset and includes larger, more chemically diverse molecules.

**Table 1:** Contents and units of each LMDB entry.

Field Name	Description	Units
atomic_numbers	Atomic numbers of all atoms	—
positions	Cartesian coordinates of all atoms	Å
energy	Total single point energy	eV
forces	Cartesian atomic forces	eV/Å
hessian	3*N* × 3*N* Hessian matrix	eV/Å^2^

All structures are stored with full Hessian matrices, including those with imaginary vibrational modes, enabling accurate benchmarking of second-order property prediction and transition state optimization. An overview of the three LMDB files is provided in Table 2.

**Table 2:** Overview of HORM LMDB files.

File Name	Subset Type	Source	# Structures	Notes
ts1x_hess_train.lmdb	Training	Transition1x	1,725,362	MLIP training data
ts1x-val.lmdb	In-distribution test	Transition1x	50,844	Eval. on similar reactions
RGD1.lmdb	Out-of-distribution test	RGD1	60,000	Eval. on larger diverse reactions

All LMDB files are compatible with Python-based readers. The code for training and testing the dataset proposed in this paper is available at the following link: https://github.com/deepprinciple/HORM/releases/tag/v1.0.

## Technical Validation

### Dataset Overview

#### Chemical space coverage

**Fig. 1:**
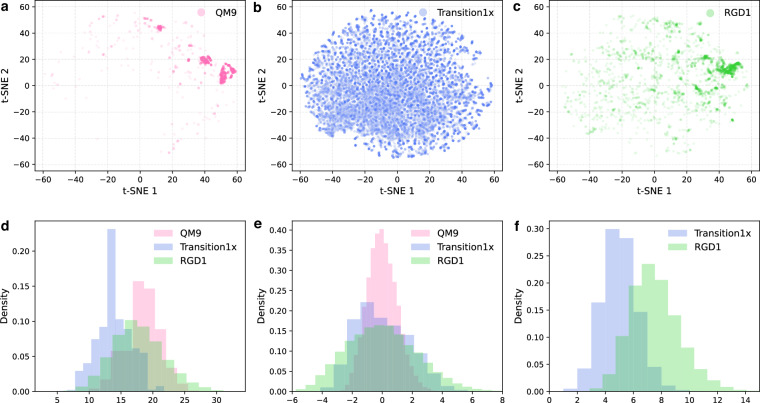
Molecular structure and property distributions of Hessian datasets. (**a**–**c**) t-SNE visualizations of MACE-OFF23-medium embeddings for 5% of sampled geometries from Hessian-QM9, HORM-Transition1x, and HORM-RGD1. (**d**–**f**) Distributions of number of total atoms, atomization energies, and number of imaginary frequencies for Hessian-QM9, HORM-Transition1x, and HORM-RGD1.

5% of geometries from Hessian-QM9, HORM-Transition1x, and HORM-RGD1 are sampled and embedded using the pre-trained MACE-OFF23 model^25^ for t-SNE visualization (Fig. 1a–c). Compared to Hessian-QM9, HORM spans a significantly broader region of chemical space. This expanded diversity is primarily attributed to the HORM-Transition1x subset, which contains approximately 40 times more molecular geometries than Hessian-QM9 and includes a wide variety of non-equilibrium structures. The HORM-RGD1 subset forms a distinct distribution with minimal overlap with HORM-Transition1x (Fig. 1c). The minimal overlap between Transition1x and RGD1 reflects their distinct generation strategies and highlights a limitation in terms of continuous coverage of chemical space. While this ensures diversity and reduces redundancy, it also means that the dataset does not provide smooth interpolation across all regions of chemical space. Expanding HORM to include additional datasets or systematically sampled intermediates would help improve continuity in future versions.

#### Properties distribution

Compared to Hessian-QM9, the data points in the HORM dataset are, on average, smaller in molecular size—with Transition1x limited to molecules containing up to seven heavy atoms—but show a markedly broader distribution in the HORM-RGD1 subset (Fig. 1d). The significantly wider range of atomization energies in HORM (Fig. 1e) reflects its coverage of a much larger portion of the potential energy surface (PES), in contrast to the equilibrium-only structures of Hessian-QM9. The distribution of imaginary vibrational frequencies (Fig. 1f) further highlights HORM’s extensive sampling of non-equilibrium states. Notably, HORM-RGD1 exhibits even broader distributions than HORM-Transition1x in both atomization energies and the number of imaginary frequencies, indicating greater deviations from equilibrium and offering a more challenging benchmark for evaluating model generalization.

### Energy, Force and Hessian Prediction

This experiment evaluates different training strategies for energy and force prediction while assessing model generalization. We investigate the effect of incorporating Hessian supervision by benchmarking models trained with energy, force, and Hessian losses (E-F-H, Eq. (2)) against those trained with only energy and force losses (E-F, Eq. (1)). All models are trained on the HORM-Transition1x training set and evaluated on both the in-distribution (ID) Transition1x validation set and the out-of-distribution (OOD) HORM-RGD1 subset.

In-distribution performance (Table 3) reflects the models’ ability to learn from the training data. For both autograd and direct-force architectures, incorporating Hessian supervision consistently improves performance across nearly all evaluation metrics. In autograd-based models, the addition of Hessian loss reduces the energy MAE by up to 25%, while resulting in minimal changes in the force MAE. However, it significantly improves second-order properties: Hessian and corresponding eigenvalue MAEs decrease by 59% and 78%, respectively.

**Table 3:** Comparison of errors for energy, force, and Hessian predictions on the HORM-Transition1x validation set.

Type	Model	Energy (eV)	Force (eV/Å)	Hessian (eV/Å^2^)	Eigenvalues (eV/Å^2^)
Autograd-based	AlphaNet (E-F)	0.044(0.028)	0.040(0.026)	0.433(0.344)	3.693(2.915)
	AlphaNet (E-F-H)	**0.034(0.013)**	0.040(0.026)	0.303(0.215)	2.332(1.555)
	LEFTNet (E-F)	0.047(0.033)	0.037(0.025)	0.366(0.302)	3.110(2.527)
	LEFTNet (E-F-H)	0.035(0.017)	**0.036(0.025)**	**0.151(0.107)**	**0.680(0.389)**
Direct-force	LEFTNet-df (E-F-H)	0.050(0.027)	0.044(0.030)	0.197(0.143)	0.680(0.389)
	LEFTNet-df (E-F)	0.054(0.033)	0.029(0.019)	1.648(1.503)	12.438(12.147)
	EquiformerV2 (E-F)	0.045(0.026)	0.021(0.014)	2.231(2.071)	20.795(21.184)
	EquiformerV2 (E-F-H)	**0.019(0.011)**	**0.016(0.010)**	**0.075(0.047)**	**0.292(0.097)**

Mean absolute errors are shown with median in parentheses. Bold values highlight the best-performing model in each task, separately for autograd-based and direct-force categories.

Direct-force models benefit more substantially from Hessian supervision, especially in the case of EquiformerV2, which achieves MAE reductions of 58% in energy, 24% in force, 97% in Hessian, and 99% in eigenvalue predictions. These results highlight the value of incorporating second-order information in directly enhancing the learned PES. Crucially, even though stochastic row sampling avoids full Hessian reconstruction, adding explicit Hessian loss markedly improves the symmetry of predicted Hessians—reducing asymmetry error (Eq. S1) by up to 94% (Table S1). This leads to more physically consistent Hessian matrices, which are essential for robust and reliable TS optimization.

Out-of-distribution performance (Table 4) evaluates the models’ generalization capabilities on unseen data. Similar patterns to those found in the in-distribution setting are observed. Autograd-based models show limited improvement in energy and force predictions but achieve notable gains in second-order properties, with Hessian and eigenvalue MAEs reduced by up to 43% and 64%, respectively. Among all models, the EquiformerV2 E-F-H variant—a representative direct-force architecture—not only achieves the largest reductions in prediction error (45%, 50%, 93%, and 97% for energy, force, Hessian, and eigenvalue, respectively), but also emerges as the best-performing model overall. Moreover, the consistently larger improvements in out-of-distribution performance compared to in-distribution performance underscore the critical role of incorporating Hessian supervision during training in enhancing model generalization.

**Table 4:** Comparison of errors for energy, force, and Hessian predictions on the HORM-RGD1 subset.

Type	Model	Energy (eV)	Force (eV/Å)	Hessian (eV/Å^2^)	Eigenvalues (eV/Å^2^)
Autograd-based	AlphaNet (E-F)	0.257(0.182)	0.151(0.131)	0.515(0.456)	5.150(4.762)
	AlphaNet (E-F-H)	0.259(0.184)	0.148(0.128)	0.415(0.360)	3.887(3.498)
	LEFTNet (E-F)	0.242(0.174)	0.132(0.114)	0.426(0.379)	4.081(3.790)
	LEFTNet (E-F-H)	**0.226(0.159)**	**0.130(0.112)**	**0.244(0.201)**	**1.458(1.166)**
Direct-force	LEFTNet-df (E-F-H)	0.304(0.224)	0.142(0.122)	0.290(0.243)	1.263(1.069)
	LEFTNet-df (E-F)	0.322(0.244)	0.146(0.128)	0.979(0.854)	4.859(4.373)
	EquiformerV2 (E-F)	0.243(0.171)	0.111(0.087)	1.224(1.047)	10.300(9.523)
	EquiformerV2 (E-F-H)	**0.133(0.089)**	**0.056(0.038)**	**0.092(0.071)**	**0.292(0.292)**

Mean absolute errors are shown with median in parentheses. Bold values highlight the best-performing model in each task, separately for autograd-based and direct-force categories.

### Transition State Search Performance

**Fig. 2:**
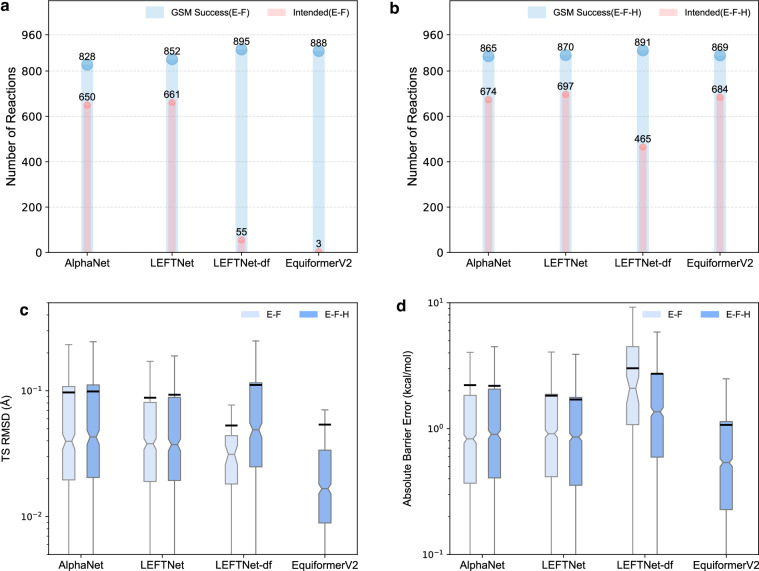
Radar plots comparing the performance of E-F and E-F-H models across different MLIPs. The six axes represent in-distribution(ID) and out-of-distribution(OOD) MAE of Hessian(H) and energies plus forces(E+F), number of intended TS and barrier prediction error. All metrics are normalized to a 0-100 scale where higher values indicate better performance. Four models evaluated are (**a**) AlphaNet (**b**) LEFTNet (**c**) LEFTNET-df and (**d**) EquiformerV2.

To evaluate the practical capabilities of reactive MLIPs in realistic TS search scenarios, we assess their performance using our recently developed end-to-end TS search workflow^34^. Four key metrics are used for benchmarking: (1) the number of successful GSM calculations, (2) the number of intended TSs—defined by whether the TS connects to the correct reactants and products after the IRC verification, (3) the root-mean-square displacement (RMSD) of optimized TS structures, and (4) the mean absolute error (MAE) of predicted barrier heights, calculated as the energy difference between the MLIP-optimized TS and the reactants (Fig. 2). GSM success reflects the model’s ability to capture the broader region around the minimal energy pathway (MEP), while the intended TS metric assesses both the quality of the initial guess and the correctness of the local curvature. TS RMSD and barrier MAE quantify the structural and energetic accuracy of the predicted TSs, both of which are essential for reliable kinetic modeling.

These four metrics collectively demonstrate that incorporating Hessian information during training (E-F-H) significantly enhances TS search performance. Among all evaluated metrics, the number of intended TSs showed the most substantial improvement, with EquiformerV2 increasing from just 3 intended TSs under E-F to 684 under E-F-H (Fig. 2a,b). While the gains observed in autograd-based models were more modest, they remain meaningful given the already strong performance of corresponding E-F models, as demonstrated by LEFTNet, which showed a  ~5% increase in intended TSs and identified the highest number (697) of intended TSs. Barrier prediction accuracy improved consistently across models, with reductions in barrier MAE of up to 10% (Fig. 2d). In contrast, TS RMSD and GSM success rates exhibited minimal improvement, as the Hessian information primarily refines the local curvature of the potential energy surface near the TS, while the TS geometry and GSM calculations are relatively insensitive to marginal improvements in energy and force predictions. Among all evaluated models, EquiformerV2 (E-F-H) achieved the best results in TS RMSD and barrier prediction, with a median TS RMSD of 0.017 Å and a median barrier MAE of 0.538 kcal/mol (Fig. 2c,d). Overall, these results underscore the critical role of Hessian information in improving both the robustness and accuracy of ML-based TS prediction.

**Fig. 3:**
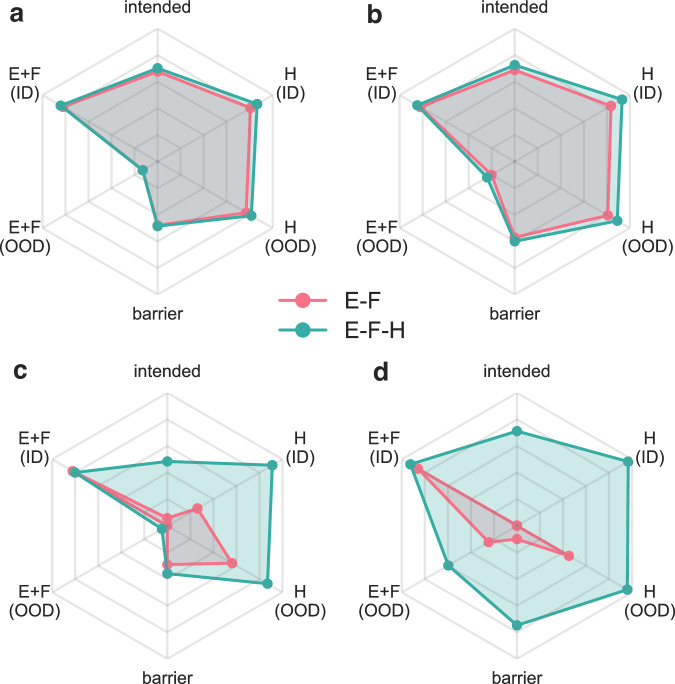
Comparison of MLIP performance in transition state search. (**a**,**b**) Number of reactions with a converged GSM pathway (blue) and intended TS (red) with different MLIP models. (**c**) Root mean square deviation and (**d**) absolute barrier error to evaluate the quality of intended TSs.

The overall performances of the four models under E-F and E-F-H loss functions are summarized in four radar plots (Fig. 3), which conclude two key general observations:

#### Consistent performance improvement

The larger hexagonal regions of the E-F-H models (green) compared to the E-F models (pink) highlight the substantial benefits of incorporating Hessian supervision, indicating enhanced predictive accuracy and generalizability. Even for autograd-based architectures (Fig. 3a,b), which already demonstrate strong baseline performance, E-F-H training yields notable improvements in both in-distribution and out-of-distribution Hessian predictions, as well as TS search performance.

#### Breakthrough improvements for direct-force architectures

While direct-force models have traditionally excelled in energy and force predictions, their application in TS search and long-time scale molecular dynamics simulations has been limited^34,45^. Here, we demonstrate that E-F-H training can substantially enhance the capabilities of direct force architectures. This is particularly evident in EquiformerV2 (Fig. 3d), which matches LEFTNet—the previously reported best-performing MLIP in TS search across seven MLIP architectures^34^. In addition, the E-F-H EquiformerV2 excels in barrier prediction and exhibits remarkable generalization ability, as evidenced by substantial improvements in the E+F(OOD) and H(OOD) metrics. This can be understood by the fact that MLIPs learn a more exact curvature of PES as more higher-order information is included.

## Supplementary information

Supplementary Information PDF file

## Data Availability

The HORM dataset is freely available at Zenodo^44^.

## Code availability

The code for training and testing the dataset proposed in this paper is available at the following link: https://github.com/deepprinciple/HORM/releases/tag/v1.0.
