# Supplementary material for: A Large Scale Molecular Hessian Database for Optimizing Reactive Machine Learning Interatomic Potentials
Source: Sci Data. 2025 Dec 4;13:37. doi: 10.1038/s41597-025-06350-5 (PMC12808639; doi:10.1038/s41597-025-06350-5)
Supplement: Supplementary file 1 — Supplementary Information PDF file [file 41597_2025_6350_MOESM1_ESM.pdf]

Supplementary Information for A Molecular Hessian Database for Optimizing Reactive Machine Learning Interatomic Potentials

Taoyong Cui[1,†], Yunhong Han[1,†], Haojun Jia[1,*], Chenru Duan[1,*], and Qiyuan Zhao[1,*]

[1]Deep Principle Inc., Cambridge, MA, 02139, USA
[†]Equal contribution.
[*]corresponding author(s): Haojun Jia (haojunjia@deepprinciple.com), Chenru Duan (duanchenru@gmail.com), Qiyuan Zhao (zhaoqiyuan@deepprinciple.com)

Hessian Asymmetry Error.

In principle, a Hessian matrix should be symmetric. However, in direct-force models, the predicted forces are non-conservative because they are not derived as gradients of a scalar energy function. As a result, the Hessians obtained by differentiating these forces are inherently asymmetric. This asymmetry can directly impact saddle point optimization. To quantify it, we define the asymmetry error as the mean absolute difference between the Hessian and its transpose:

$$\frac{1}{N^2}\sum_{i,j=1}^{N}\left|H_{ij}-H_{ji}\right|, \tag{S1}$$

Computational details.

All calculations in this study were conducted on the Volcengine computing platform. Transition state (TS) search experiments were executed as bundled single-core jobs on a CPU node equipped with 32 effective cores (Intel Cascade Lake, 2.40 GHz) and 128 GB of memory. Density functional theory (DFT) calculations employed GPU4PYSCF[1] as the quantum chemistry engine and were run on NVIDIA A30 GPU cards. MLIP training was also performed on A30 GPUs and H20 GPUs.

Hyperparameters.

In this work, we evaluate three distinct models: AlphaNet, LeftNet (with both autograd and direct-force variants), and EquiformerV2. The training batch size is selected from {8, 16, 32}, depending on the available GPU memory to ensure efficient utilization. AlphaNet employs a 4-layer architecture with a hidden dimension of 128 and 16 attention heads, designed to improve its representational capacity. Both the autograd and direct-force variants of LeftNet are configured with a deeper 9-layer architecture and a larger hidden dimension of 256 to support more complex learning dynamics. EquiformerV2 uses a 4-layer architecture with a hidden dimension of 128, 4 attention heads, and a maximum spherical harmonic degree of $l_{max} = 4$, and 4 attention heads. As mentioned earlier, to reduce computational overhead, we randomly sample a subset of columns from each Hessian matrix during training. The number of sampled columns—referred to as the number

of reference Hessian rows (N_{HR})—is set to 1 for autograd-based models and 2 for direct-force-based models. These hyper-parameters are summarized in the Table S3.

Supplementary Tables

Table S1: Comparison of Hessian Asymmetry Error (eV/Å^2) between HORM-Transition1x validation set and HORM-RGD1 subset.

**Model**	**Transition1x-val**	**RGD1**
LEFTNet-df(E-F)	1.447 (1.271)	1.184 (1.033)
LEFTNet-df(E-F-H)	0.211 (0.163)	0.290 (0.253)
EquiformerV2(E-F)	1.235 (1.153)	1.071 (0.861)
EquiformerV2(E-F-H)	0.073 (0.055)	0.092 (0.075)

Table S2: Hyperparameters used for training MLIP models.

**Model**	**Layers**	**Hidden Dim**	**Heads**	$\mathbf{N_{HR}}$	**Learning Rate**	**Batch Size**
AlphaNet	4	128	16	1	1×10^{-4}	32
LeftNet (ag)	9	256	NA	1	5×10^{-5}	64
LeftNet (df)	9	256	NA	2	5×10^{-5}	64
EquiformerV2	4	128	4	2	3×10^{-4}	128

References

[1] Xiaojie Wu, Qiming Sun, Zhichen Pu, Tianze Zheng, Wenzhi Ma, Wen Yan, Xia Yu, Zhengxiao Wu, Mian Huo, Xiang Li, et al. Python-based quantum chemistry calculations with GPU acceleration. *arXiv preprint arXiv:2404.09452*, 2024.
